# Possibilities of Transcatheter Intracerebral Laser Photobiomodulation Therapy in Patients with Dementia in the Treatment of Various Microcirculatory Cerebral Lesions

**DOI:** 10.1002/alz.087886

**Published:** 2025-01-09

**Authors:** Ivan V Maksimovich

**Affiliations:** ^1^ Clinic of Cardiovascular Diseases named after Most Holy John Tobolsky, Moscow, Moscow Russia

## Abstract

**Background:**

Most cerebrovascular lesions are aggravated by dementia. This study examines the possibility of reducing dementia by stimulating cerebral angiogenesis and neurogenesis using Transcatheter Intracerebral Laser Photobiomodulation Therapy (PBMT) in patients with Alzheimer’s disease (AD), distal cerebral atherosclerosis, Binswanger’s disease (BD), and vascular parkinsonism (VP).

**Methods:**

The study included 404 patients with dementia, aged 29‐81 (mean age 78).

Examination: assessment of CDR, TDR, MMSE, cerebral MRI, MRA, CT, MSCTA, scintigraphy (SG), rheoencephalography (REG), cerebral multi‐gated angiography (MUGA).

48 (11.88%) patients suffered from AD. According to dementia severity, patients were divided into: preclinical stage (TDR‐0) ‐ 4, mild stage (TDR‐1) ‐ 16, moderately severe stage (TDR‐2) ‐ 21, severe stage (TDR‐3) ‐ 7 patients.

356 (88.12%) patients suffered from diseases such as distal cerebral atherosclerosis ‐ 302 (84.83%), BD ‐ 17 (4.78%), VP ‐ 37 (10.39%) people. According to dementia severity, patients were divided into: unnoticed dementia ‐ 21, dementia at level (CDR‐1) ‐ 312, (CDR‐2) ‐ 23 people.

All patients underwent Transcatheter Intracerebral Laser Photobiomodulation Therapy (PBMT).

**Results:**

All 48 (100%) AD patients showed improvement in cerebral microcirculation and 10‐20% increase in cerebral temporal lobes volume, which indicates the development of cerebral angiogenesis and neurogenesis as well as tissue regeneration. The process was accompanied by dementia level decrease and cognitive functions restoration. Consequently, patients were transferred to a milder TDR group. Depending on the initial dementia severity, the resulting positive effect is observed for 2‐12 years.

All patients with distal cerebral atherosclerosis 302 (100%), BD 17 (100%) and VP 27 (100%) after Transcatheter Intracerebral Laser Photobiomodulation Therapy (PBMT) showed improved cerebral microcirculation, decreased cerebral involutional changes, which indicates the development of angiogenesis, neurogenesis, and tissue regeneration. This led to a long‐term (over 10 years) decrease in dementia level and cognitive functions restoration.

**Conclusions:**

Despite the differences in AD etiology, distal cerebral atherosclerosis, BD and VP, Transcatheter Intracerebral Laser Photobiomodulation Therapy (PBMT) is an effective method for stimulating angiogenesis and neurogenesis. It leads to improved cerebral blood supply and restoration of tissue structures, dementia decrease, and mental and cognitive functions improvement. The resulting effect lasts for a long time.

